# Three-dimensional characterization of root morphology for maxillary incisors

**DOI:** 10.1371/journal.pone.0178728

**Published:** 2017-06-08

**Authors:** Carly A. Ahlbrecht, Antonio Carlos de Oliveira Ruellas, Beatriz Paniagua, Juan A. Schilling, James A. McNamara, Lucia Helena Soares Cevidanes

**Affiliations:** 1 Department of Orthodontics and Pediatric Dentistry, School of Dentistry, University of Michigan, Ann Arbor, Michigan, United States of America; 2 Department of Orthodontics and Pediatric Dentistry, Federal University of Rio de Janeiro, Rio de Janeiro, Rio de Janeiro, Brazil; 3 Department of Psychiatry, University of North Carolina at Chapel Hill, Chapel Hill, North Carolina, United States of America; University of Kentucky, UNITED STATES

## Abstract

The aim of this study was to test the reproducibility of three-dimensional (3D) surface models of maxillary incisors and to propose a characterization of root morphology. The sample was comprised of pre-treatment cone-beam computed tomography (CBCT) images of fifty-five patients. The CBCTs were used to construct 3D surface models of the maxillary incisors. The reproducibility of surface models was tested by repeated construction of them by two observers. A 3D surface model that corresponded to the average of all lateral and all central incisors was generated. 3D surface distances and vector differences were calculated for each individual tooth and the average of the teeth considered. The corresponding points on the 3D surface mesh for each subgroup were compared statistically to those of the neutral subgroup using shape analysis MANCOVA and Hotelling’s t-statistic (p < 0.05). Repeated construction of surface models demonstrated adequate inter-rater reproducibility. The distribution of 3D models into root morphology subgroups was: blunt (11% and 26% of the central and lateral incisors, respectively), conical (15% of the central incisors), long (27% and 20% of the central and lateral incisors, respectively), and short (15% and 4% of the central and lateral incisors, respectively). Compared to the neutral average, statistically significant differences in root morphology were found for blunt, long, conical, and short central incisors and for blunt, long, and short lateral incisors. We can conclude that 3D surface models construction for upper incisors is reproducible. 3D shape analysis using CBCT images allows a phenotypic characterization of incisor root morphology.

## Introduction

External apical root resorption (RR) is present in 7% to 13% of individuals who have not had orthodontic treatment [1,2] An increase in RR, primarily in the maxillary anterior teeth, is a commonly occurring pathological sequela of orthodontic treatment [3]. Greater than 2 mm of RR in 25% of orthodontic patients[4] or even >3 mm in 30% of patients[5] has been reported. Mechanical forces and other environmental factors do not explain adequately the variation seen among individual expressions of RR[6].

Root resorption susceptibility following the application of an orthodontic force can be associated with tooth type (with a greater risk for the upper incisor)[7], treatments with extraction[7–9], the presence of supraocclusion or open bite[7], treatment duration[10], root resorption before treatment[8,9], and root morphology[7–9]. Although root resorption can occur with or without orthodontic treatment, research in this area indicates that RR is influenced by a complex genetic trait[3,6,11,12], individual predisposition and multifactorial etiology[4,12–16]. Orthodontic patients with sharp, pointed (triangle-shaped) roots that require premolar extraction, however, have a greater risk of severe root resorption[17]. Therefore, interest in variability in root morphology has increased recently[18,19].

Maxillary incisor root morphology has been characterized in several studies in the orthodontic literature[5,20,21]. These efforts have been qualitative and were based on two-dimensional (2D) radiographic observations. Levander and Malmgren[20], and later Mirabella and Årtun[21] described and schematically illustrated classification systems for maxillary incisor root morphology that have been applied in additional studies[4,22,23]. These subjective 2D interpretations of root morphology describe normal, blunt, eroded, bent, pointed, or bottle (pipette) incisor root shapes. Taithongchai et al.[24] presented a 2D quantitative evaluation of tapering of incisor root form. To date, efforts to categorize maxillary incisor root morphology have not utilized three-dimensional (3D) shape analysis techniques.

Previous studies using cone-beam computed tomography (CBCT) have demonstrated high accuracy and reliability of measurements of root length and root shortening when CBCT images are compared both to direct skull measurements[25] and to periapical radiographs[26]. A recent case report using CBCT, microfocus CT and scanning electron microscopy describes the valuable use of CBCT in the diagnosis of root resorption[27]. Furthermore, *ex-vivo* studies using bovine teeth showed that, when compared to measurements made from micro CT, CBCT scans detect simulated defects of 0.6 mm depth accurately[28].

With the growing use and improved quality of CBCT images, recent research has focused on evaluating the use of these images for detection and quantification of external apical root resorption (EARR), which includes resorption that is induced orthodontically (OIIRR)[29]. It has been proposed that the enhanced root visualization afforded by CBCT could be important in the assessment of pre-orthodontic and post-orthodontic root status[30]. Additionally, the ability of CBCT to provide distortion-free, slice-by-slice views of individual tooth roots offers an excellent opportunity to evaluate root resorption[25].

For these reasons, the current study utilizes CBCT images for a novel characterization of 3D incisor root morphology, applying a 3D surface mapping technique. This new technique of shape analysis has been applied previously for assessment of brain morphometry in MRI and for localization and quantification of the extent of resorptive changes in mandibular condyles using CBCT images[31–33]. The present study is the first to apply this method to characterize incisor root morphology.

This study was conducted to validate the reproducibility of the construction of 3D surface models of maxillary incisors and to test a novel and detailed phenotypic characterization of pre-treatment root morphology.

## Methods

### Sample

Data collection for this study was approved by the University of Michigan Health Sciences Institutional Review Board (#HUM00064320).

CBCT baseline scans of 112 consecutively-treated patients from two private orthodontic practices (Practices A and B) were utilized. Twenty-second CBCT scans using a Classic i-Cat machine (Imaging Sciences International, Hatfield, PA) were taken using 0.3 mm^3^ voxel size (120 kVp, 5mA) by Practice A and 0.4 mm^3^ voxel size (120 kVp, 28.3 mA) by Practice B. The following inclusion criteria were applied to the sample:

1. Subjects must have been 10–18 years old at the onset of treatment and have a pre-treatment CBCT of sufficient quality to be segmented accurately.
2. Subjects must have complete root formation of the maxillary right central and lateral incisors at the time of the initial scan.
3. Subjects must be free from periapical pathology at the maxillary right central and lateral incisors.

The exclusion criteria were:

1. Subjects with unerupted canines in close proximity to the root apex of the maxillary right central or lateral incisor.
2. Subjects having undergone previous orthodontic treatment.
3. Subjects with previous endodontic or restorative treatment of the maxillary right central or lateral incisor.

55 patients who satisfied the above criteria were included in this study, 25 from Practice A and 30 from Practice B. The average patient age was 13 years 5 months (± 1 year 5 months), with 65% females and 35% males.

### Measurement

To standardize voxel size, all scans were re-sliced to 0.3 mm^3^ voxel size using Slicer3D (open-source, http://www.slicer.org). The goal was to improve the computational power and decrease the time for image analysis.

#### Construction of 3D volumetric label maps (segmentation)

**Fig 1:**
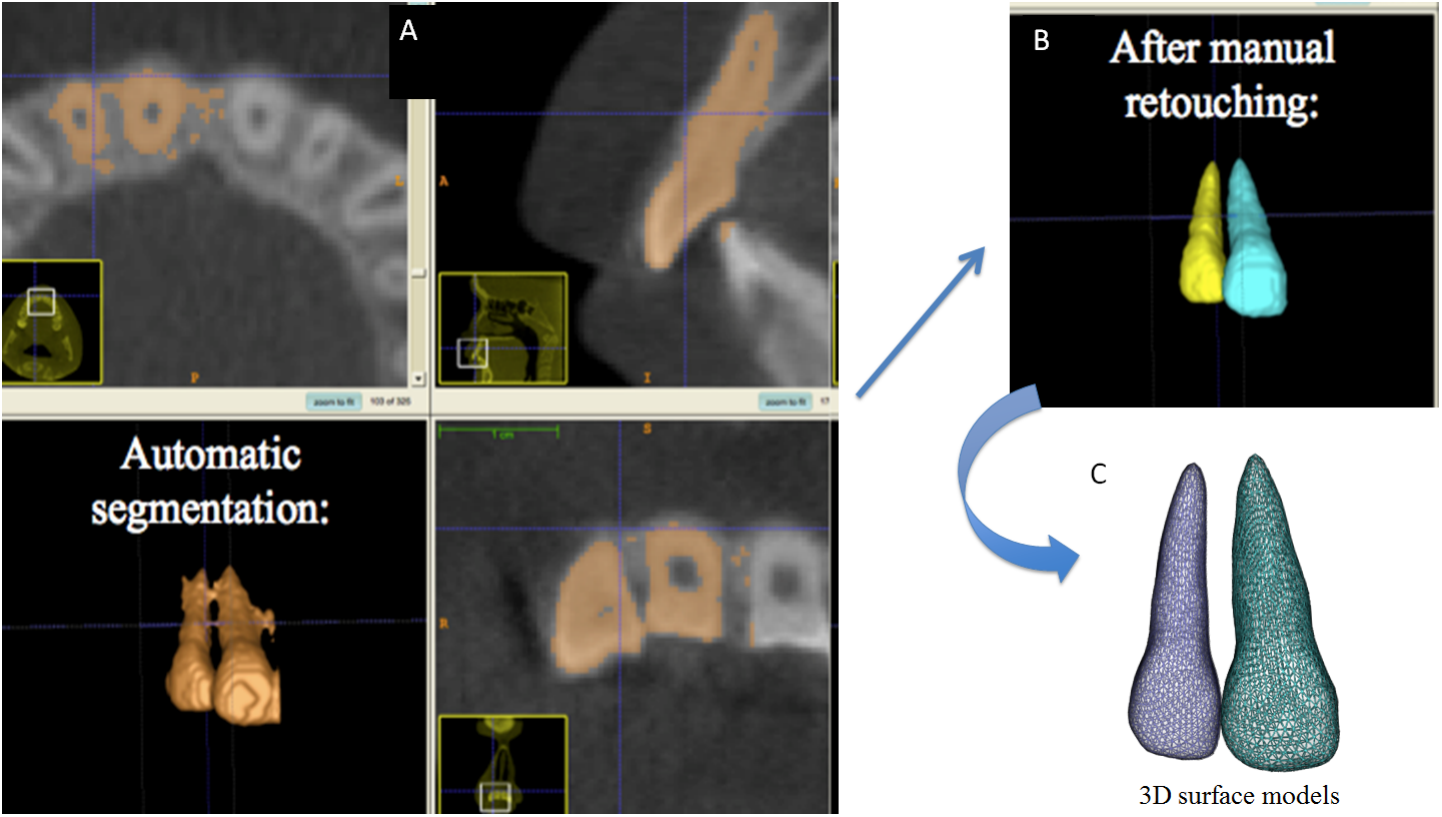
A and B) Construction of 3D volumetric label maps: resulting from automatic segmentation and after manual retouching by careful inspection of grey level boundaries in cross-sectional slices; C) 3D surface models.

The process of constructing 3D volumetric label maps from CBCT scans is called segmentation. The label maps were constructed for the right central and lateral incisors to assess the individual morphology of these teeth. All scans were segmented automatically with IntensitySegmenter software (distributed as a 3DSlicer– www.slicer.org—extension, https://www.slicer.org/slicerWiki/index.php/Documentation/Nightly/Extensions/IntensitySegmenter) and the output files were checked by a rater interactively with ITK-SNAP software (open-source software, www.itksnap.org)[34] slice by slice in all three planes of space (sagittal, coronal and axial) to correct for any errors in the automatic segmentation (Fig 1).

The 3D volumetric label maps were converted to 3D surface models in Slicer software, and the resulting surface models of individual central and lateral incisors were saved as separate 3D surface models (3D surface meshes) that described the 3D tooth shape.

#### Validation of methodology

A single investigator (CAA) constructed and refined the 3D volumetric label maps for right maxillary incisors of the 55 patients included in this study. To confirm the repeatability of the segmentation process, ten random samples were selected for repeated segmentation by a second investigator (JAS). The resulting 3D surface models then were overlaid using VAM software (Canfield Scientific, Fairfield, NJ), and the number of points, triangles, surface area, and volume of the corresponding pair of 3D surface models were computed to assess intra-class correlations. Signed distances between the two models were calculated to provide the mean, median, standard deviation, and magnitude of maximum differences between repeated surface models.

#### Standardization of coordinate system

The 3D surface models for the maxillary right central incisors were loaded in VAM software. Using a surface-to-surface best-fit alignment (automatic surface registration in VAM software using the entire surface as reference), each tooth in the sample was superimposed to establish a common coordinate system that approximated all the individual teeth within three-dimensional space.

**Fig 2:**
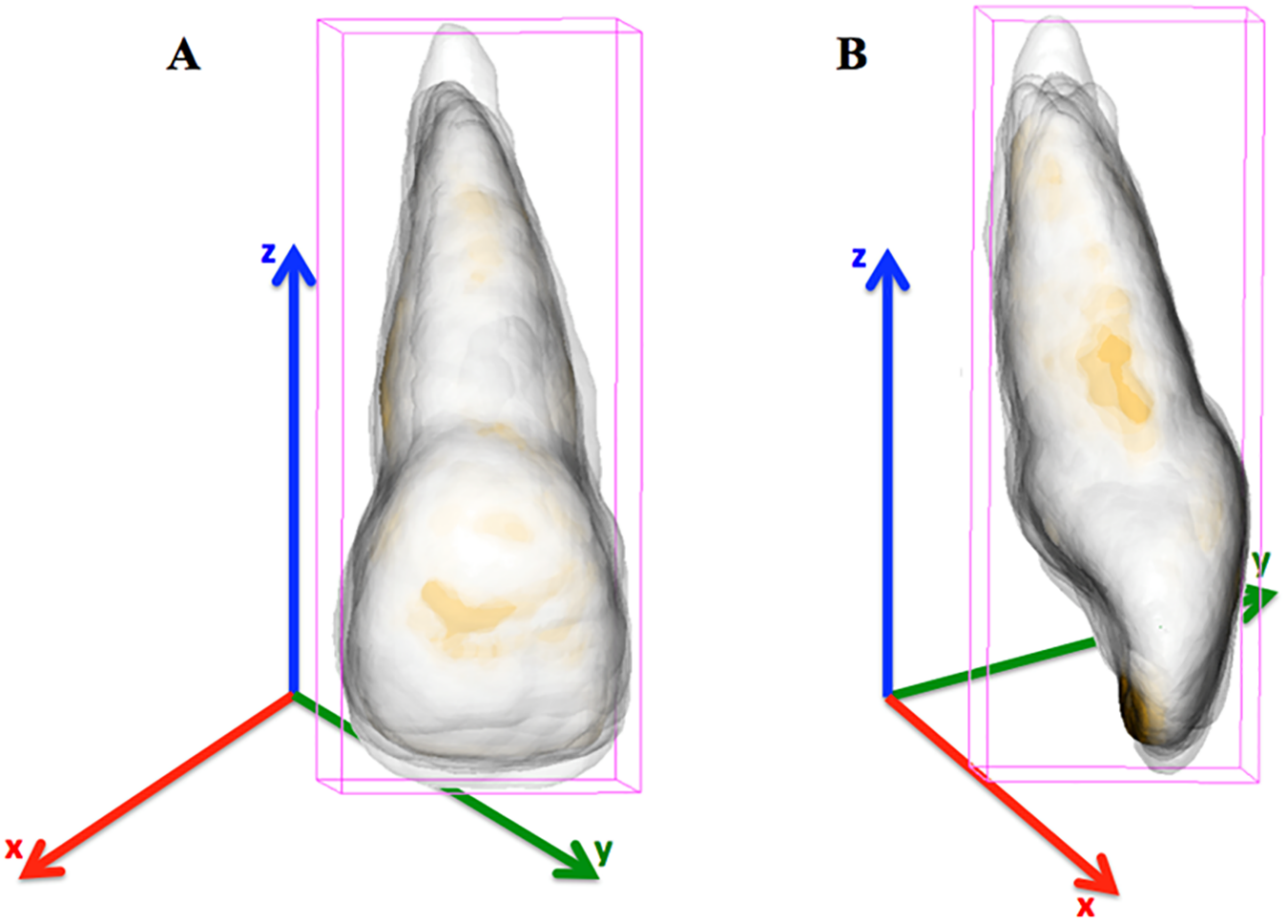
Approximation and superimposition of all central incisors 3D surface models, under the same coordinate system. Frontal (A) and distal (B) views.

The best-fit alignment utilized the entire tooth structure of each surface mesh because there was no stable structure of reference for registration. The crown of the incisor, for example, is not consistent across the sample. Approximation of the 3D image of the central incisors is shown in Fig 2.

#### Parameterization of corresponding points between all teeth

**Fig 3:**
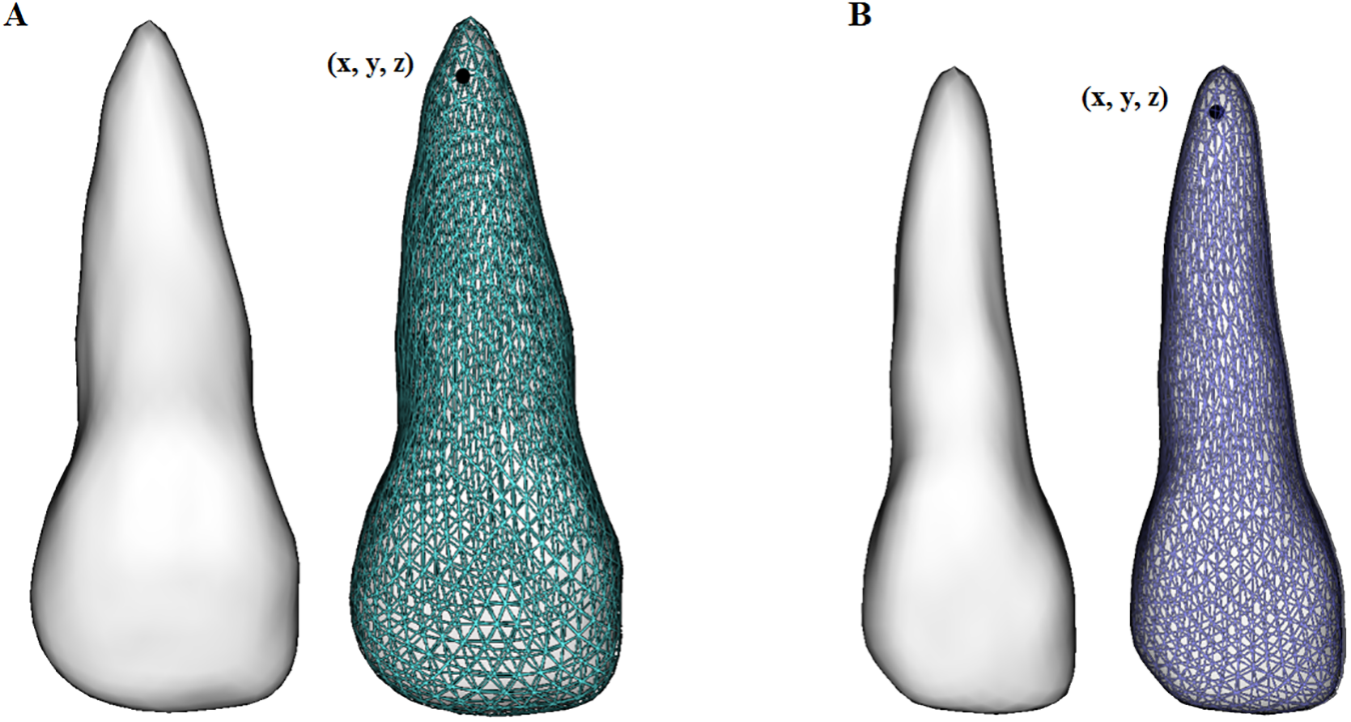
Examples of corresponding point-based surface meshes for a central incisor (A) and lateral incisor (B). Each one of the 1002 surface points (with xyz coordinates) on the mesh of the 3D surface models correspond to each other for all 55 central and lateral incisors.

SPHARM-PDM software (distributed as a 3DSlicer– www.slicer.org—extension, http://www.nitrc.org/projects/spharm-pdm) was used to compute the parameterization of 1002 corresponding surface points among all central incisors and among all lateral incisors. SPHARM-PDM computed a mesh approximation from the segmented label files, whose 1002 surface mesh points and their respective xyz coordinates were optimized for each corresponding tooth mesh (Fig 3).

#### Creating the average mesh

Linux scripts (MeshMath) distributed as part of SPHARM-PDM was used to create the average mesh for the upper right central incisor and upper right lateral incisor based on the fifty-five teeth sample. In this surface averaging process, the 1002 original surface point correspondences were propagated through all stages of deformations and were used for object averaging. The affine transformations were applied to the points individually. In geometry, an affine transformation is a transformation that preserves ratios of distances between points lying on a surface model, where parallel lines will remain parallel to each other after an affine transformation. Grouping all the mean points provided the linear and nonlinear deformation fields that resulted in the average tooth shape, which subsequently was used as reference tooth for superimposition and classification of the morphological differences.

#### Calculation of differences between each tooth and the average tooth (signed distances, absolute and vector differences)

**Fig 4:**
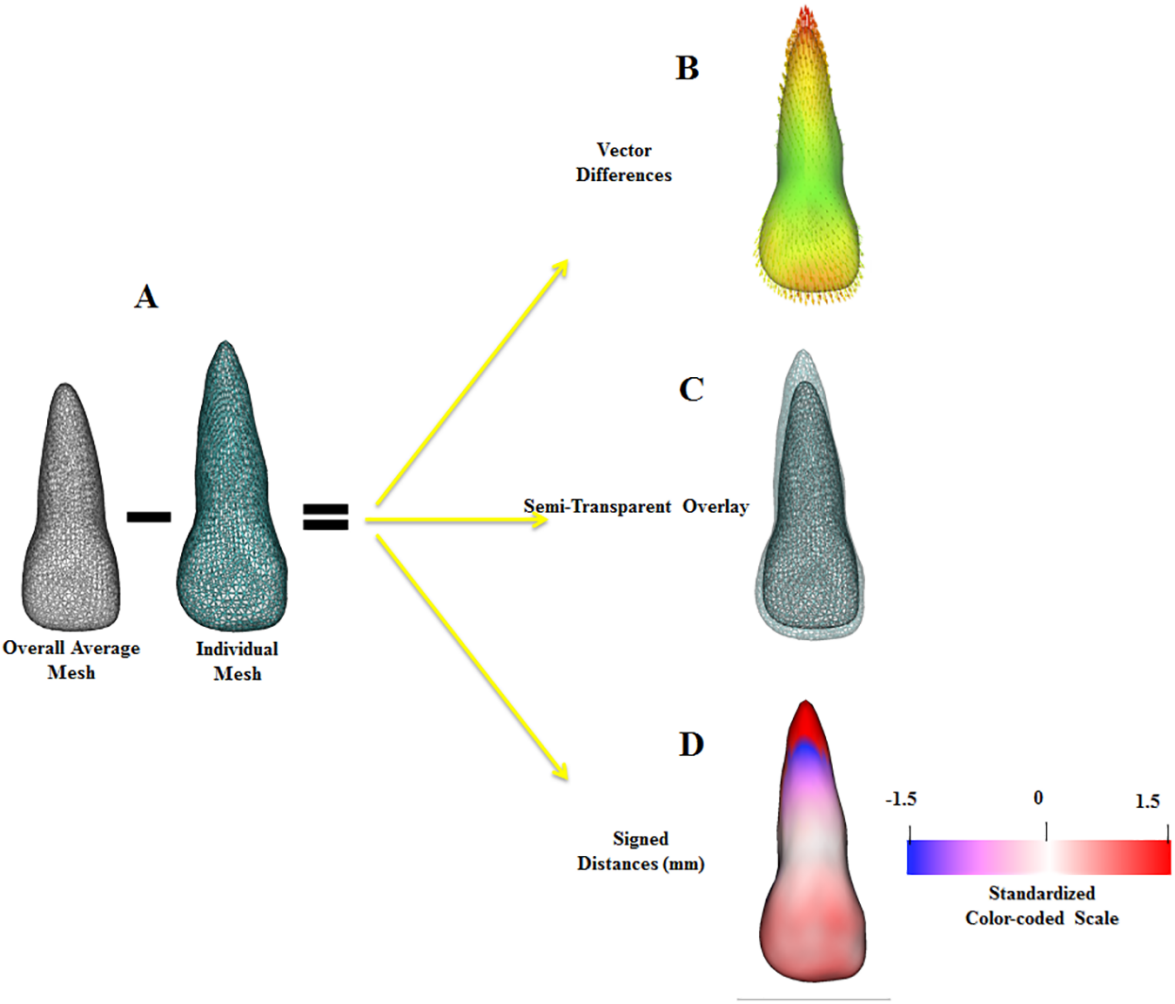
Summary of the surface-to-surface distance calculations. The 1002 corresponding surface points on the individual mesh were subtracted from the homologous points on the overall average mesh (A) to calculate vector differences (B). The dimension and directionality of the vector differences were computed as signed 3D linear distances, where blue indicated a region smaller than the average and red indicated a region larger than the average (D). A semi-transparent overlay (C) of the individual with the average 3D model was used for quality control of the signed distances calculations.

MeshMath calculated 3D differences between each individual tooth compared to the average to assess patterns of morphological differences quantitatively in the sample using 3D vector differences and signed surface distances. The computed 1002 vector differences were displayed on the 3D surface model of the tooth, one for each point on the surface mesh, scaled according to the magnitude of the difference and pointing in the direction of the change (Fig 4).

With the goal to help the visualization of the differences, the patterns of variation across the sample were determined through calculation of signed distances using the MeshMath script, where the individual surface was displayed as smaller (negative, blue), the same (0 surface distances, white), or larger (positive, red) than the average. The blue to red color-coded scale was standardized, allowing a proper comparison of all teeth within the sample. For the central incisors, pure blue was set at -1.5 mm and pure red at 1.5 mm. For the lateral incisors, pure blue was set at -3 mm and pure red at 3 mm, as morphological variability of lateral incisors was greater (Fig 4).

#### Supervised criteria for 3D morphology subgroups

**Fig 5:**
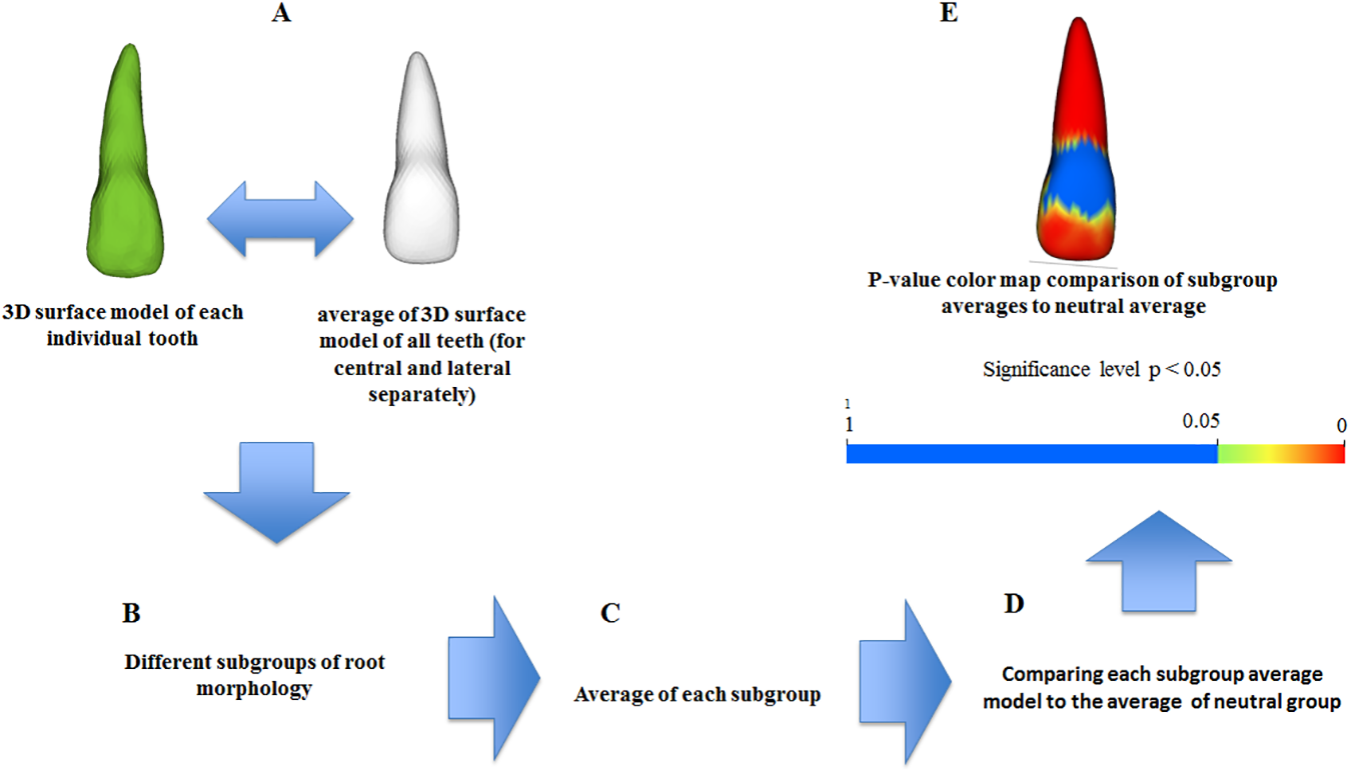
Flowchart of the steps for 3D characterization of maxillary incisor in subgroups morphology. Individual teeth were classified into a morphology subgroup (A, B). Subgroup averages were created using surface averaging of the constituent teeth (C). Regions of statistically significant morphologic variability were visualized (E) using p-value color maps (p < 0.05) comparing subgroup average models to the neutral average (D).

Subgroups of root morphology were defined quantitatively from analysis of the comparisons of individual 3D models of the root surface with the average 3D surface model of the central and lateral incisors separately (Fig 5A and 5B). Subgroup averages were created using surface averaging of the constituent teeth (Fig 5C); the morphological subgroups were determined by the calculation of root morphology signed distances. The subgroups shared phenotypic characterization following the proposed classification, with the neutral group serving as the reference group.

The definition of each root classification was adapted from previous 2D descriptions[20,21]. The following morphology subgroups were proposed:

**Neutral—**individual root that closely approximates the overall sample average
**Blunt**—root that presented apices notably shorter and less pointed, while the cervical region was wider than the overall sample average
**Long—**root larger than the overall sample average in all dimensions (length and circumference)
**Conical**—root narrower in circumference, but may or may not have increased length compared to the overall sample average
**Short**—root that presented obvious decrease in apical length and narrower circumference compared to the overall sample average
**Dilacerated**—root that presents distal, mesial or lingual dilaceration compared to the overall sample average

#### Statistical analysis

Statistical analysis was completed using shape analysis MANCOVA and Hotelling’s t-statistics (p < 0.05). This method calculated the projections of the vector directions for each tooth to determine areas statistically different from the average morphology (Fig 5D and 5E). Statistical significance was assessed using a permutation approach, where the vector was recomputed for each permutation.

Because the dimension of the data is 1002 (1002 surface points on each tooth mesh) while the sample size is only 55, this study assessed high dimensional low sample size variability. To correct for multiple comparisons, a false discovery rate estimation (FDR) at 0.05 was applied. FDR correction allows confidence that 95% of areas described as statistically significant are true positives. The FDR correction provided an interpretable and adaptive criterion with higher power than non-parametric permutation tests[35].

## Results

### Validation study

The surface-to-surface distance calculations of the repeated segmentation models indicated high reproducibility of the 3D shape analysis protocol. The average of the overall mean differences between surface models was 0.06±0.13 mm, and the maximum surface differences ranged from 0.36 to 0.86 mm. The number of surface points and triangles in the repeated meshes had excellent reproducibility, with an intra-class correlation (ICC) of 0.99; both surface area and volume of the repeated models had an ICC of 0.98.

Table 1 shows the descriptive statistics of the surface differences between all the points in the repeated surface models.

**Table 1:** Descriptive statistics of inter-observer differences computed as surface-to-surface distances between repeated incisor surface models.

Case	Tooth	Voxel (mm^3^)	Median Difference(mm)	Max Difference(mm)	Mean Difference (mm)	SD
**1**	UR1	0.3	0.06	0.59	0.06	0.15
**1**	UR2	0.3	0.03	0.51	0.04	0.14
**2**	UR1	0.4	0.04	0.40	0.05	0.12
**2**	UR2	0.4	0.00	0.64	0.02	0.13
**3**	UR1	0.3	0.00	0.55	0.00	0.12
**3**	UR2	0.3	0.00	0.66	0.04	0.13
**4**	UR1	0.4	0.03	0.55	0.05	0.14
**4**	UR2	0.4	0.00	0.50	0.06	0.13
**5**	UR1	0.4	0.08	0.67	0.08	0.17
**5**	UR2	0.4	0.05	0.86	0.05	0.17
**6**	UR1	0.3	0.08	0.49	0.10	0.12
**6**	UR2	0.3	0.07	0.49	0.09	0.10
**7**	UR1	0.3	0.03	0.43	0.04	0.09
**7**	UR2	0.3	0.01	0.36	0.01	0.10
**8**	UR1	0.4	0.15	0.54	0.14	0.15
**8**	UR2	0.4	0.10	0.50	0.11	0.11
**9**	UR1	0.4	0.08	0.56	0.10	0.13
**9**	UR2	0.4	0.10	0.50	0.11	0.11
**10**	UR1	0.4	0.02	0.53	0.03	0.12
**10**	UR2	0.4	0.00	0.40	0.01	0.10
**Total inter-observer variability:**				Range: 0.36–0.86	Average: 0.06	0.13

#### 3D phenotypic characterization of incisor morphology

**Fig 6:**
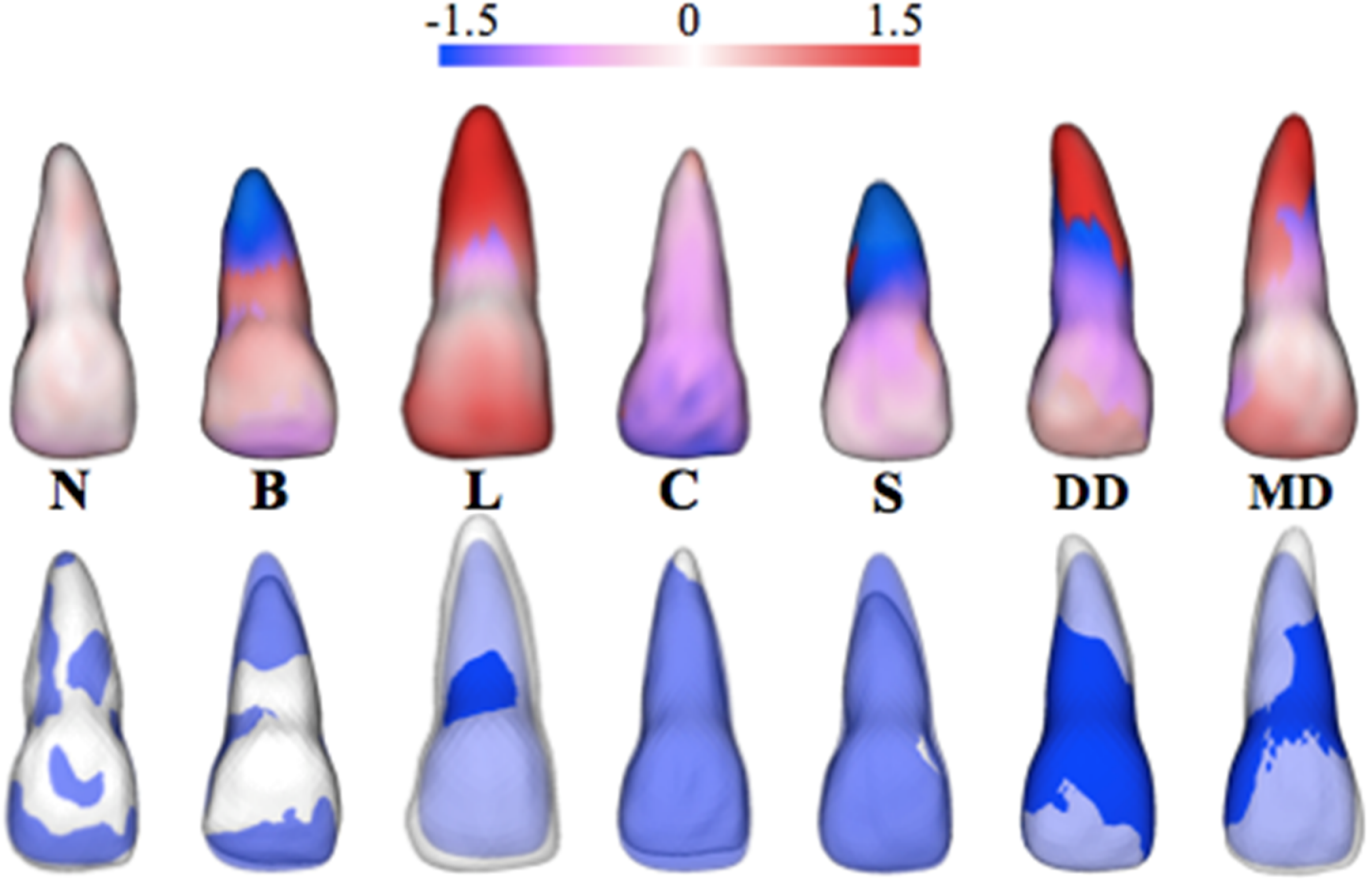
Characteristic examples of individual morphological variability. Morphological variability quantified by signed-distance color maps, standardized at ± 1.5 mm for visual comparisons (top), and semi-transparent overlays (bottom). Individual tooth examples are shown for each central incisor classification subgroup: neutral (N), blunt (B), long (L), conical (C), short (S), distal dilaceration (DD), and mesial dilaceration (MD).

Neutral, blunt, long, conical, short, distal dilaceration, and mesial dilaceration root morphologies following the proposed criteria described in the methods section were observed. A signed-distance color map and semi-transparent overlay are provided as examples of each central incisor morphology subgroup (Fig 6).

#### Sample distribution

The distribution of central and lateral incisors into subgroups is shown in Table 2.

**Table 2:** Distribution of maxillary right central and lateral incisors into morphology subgroups. Total sample size n = 55.

	**Central Incisor**		**Lateral Incisor**	
**Morphology Subgroup**	Number of Teeth	Percent of Total	Number of Teeth	Percent of Total
Neutral	14	25.5	14	25.5
Blunt	6	10.9	14	25.5
Long	15	27.3	11	20.0
Conical	8	14.5	0	0.0
Short	8	14.5	2	3.6
Lingual dilaceration	0	0.0	3	5.5
Distal dilaceration	3	5.5	9	16.4
Mesial dilaceration	1	1.8	2	3.6

#### Average tooth model for morphology subgroups

**Fig 7:**
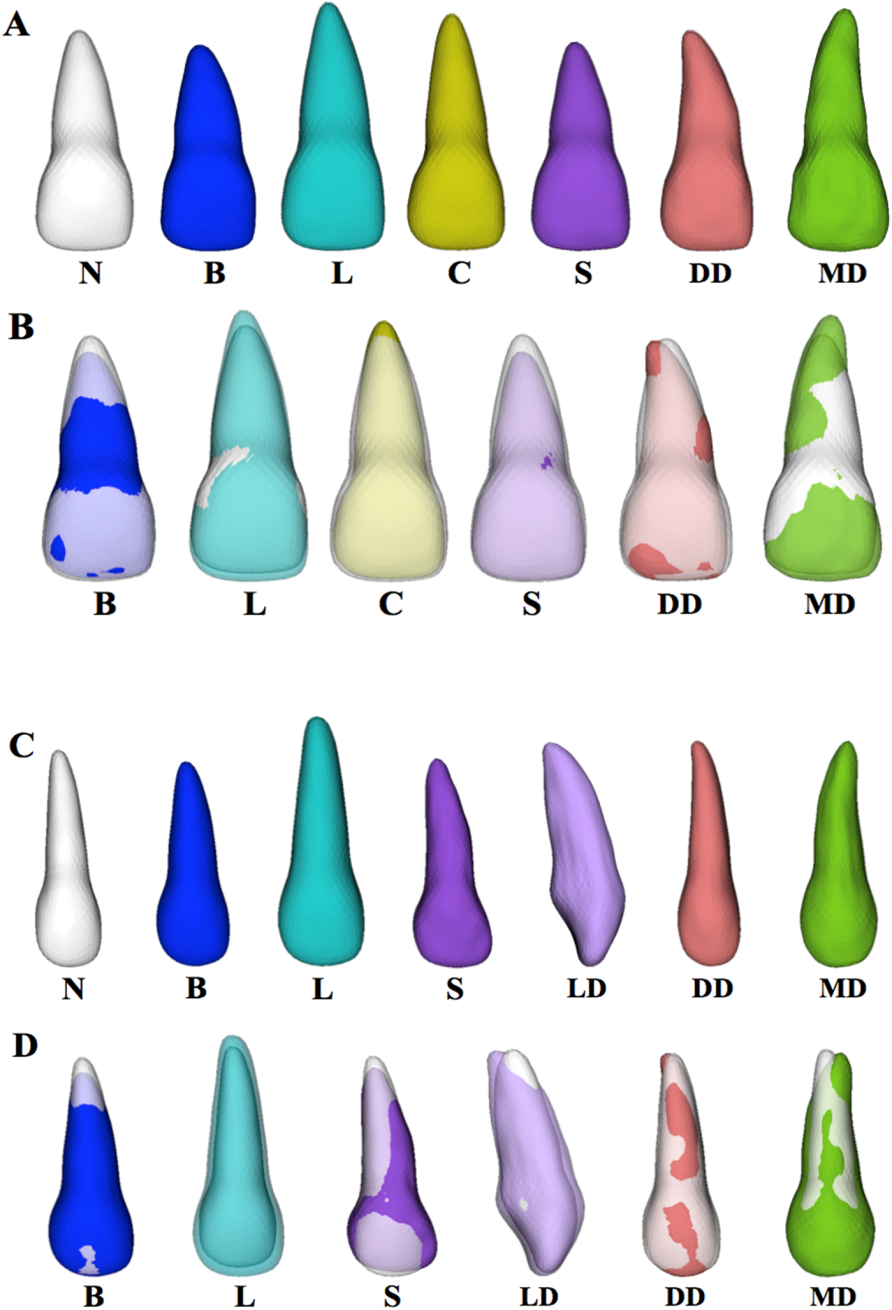
Average models for neutral (N), blunt (B), long (L), conical (C), short (S), lingual dilaceration (LD), distal dilaceration (DD), and mesial dilaceration (MD) maxillary right central and lateral incisor morphology subgroups. Neutral average is shown in white; other subgroup averages are shown in arbitrary colors for better visualization. A) Central incisor average models. B) Central incisor morphology subgroup averages overlaid with semi-transparent neutral average. C) Lateral incisor average models. D) Lateral incisor morphology subgroup averages for overlaid with semi-transparent neutral average. Lingual dilacerations model is shown from distal view for better visualization of characteristic morphology.

The averaged neutral group model was used as a standard against which the other subgroup average models were tested statistically. Each subgroup average model also was compared visually to the neutral average model using semi-transparent overlays (Fig 7).

### Statistical analysis

#### Raw p-value color maps

**Fig 8:**
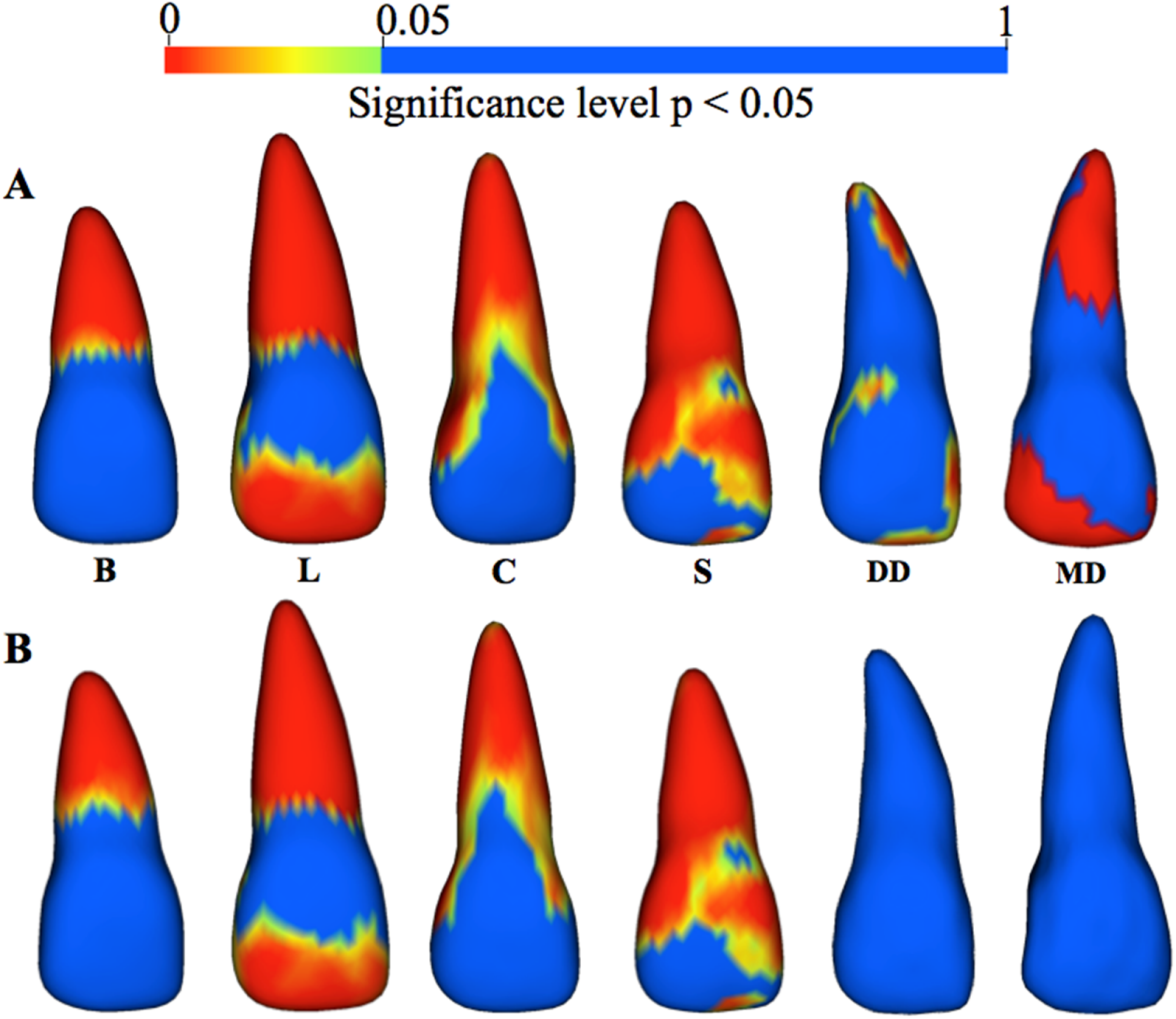
(A) Raw p-value (p < 0.05) color maps showing regions of statistically significant surface-to-surface differences for central incisor blunt (B), long (L), conical (C), short (S), distal dilaceration (DD), and mesial dilaceration (MD) subgroup average models compared to the neutral average using Hotelling’s ttests. (B) P-values corrected for false discovery rate at 0.05.

Regions of statistically significant differences (p < 0.05) in root morphology from subgroup average models compared to the neutral average were found for all central incisor subgroups using Hotelling’s t-tests. Additionally, significant differences in crown morphology also were observed for all groups except for the blunt group. Raw p-value color maps for central incisors can be found in Fig 8. The regions of significant morphological differences between the subgroup averages to the neutral average were as follows:

**Blunt**—wider at the cervical portion of the root and shorter at the apex.
**Long**—longer and wider along entire root surface.
**Conical**—narrower along entire root surface.
**Short**—narrower along entire root surface and shorter at apex.
**Distal dilaceration—**narrower at mesial aspect of apical 1/3 of root, distally deviated at root apex.
**Mesial dilaceration**—narrower at mesial aspect of apical 1/3 of root, longer and mesially deviated at apex.

With FDR at 0.05, significant differences for the mesial and distal dilaceration average models were not observed compared to the neutral average (Fig 8). The distribution of statistically significant regions for the blunt, conical, long, and short, as compared to the neutral average, were very similar to what was observed with the raw p-value color maps.

**Fig 9:**
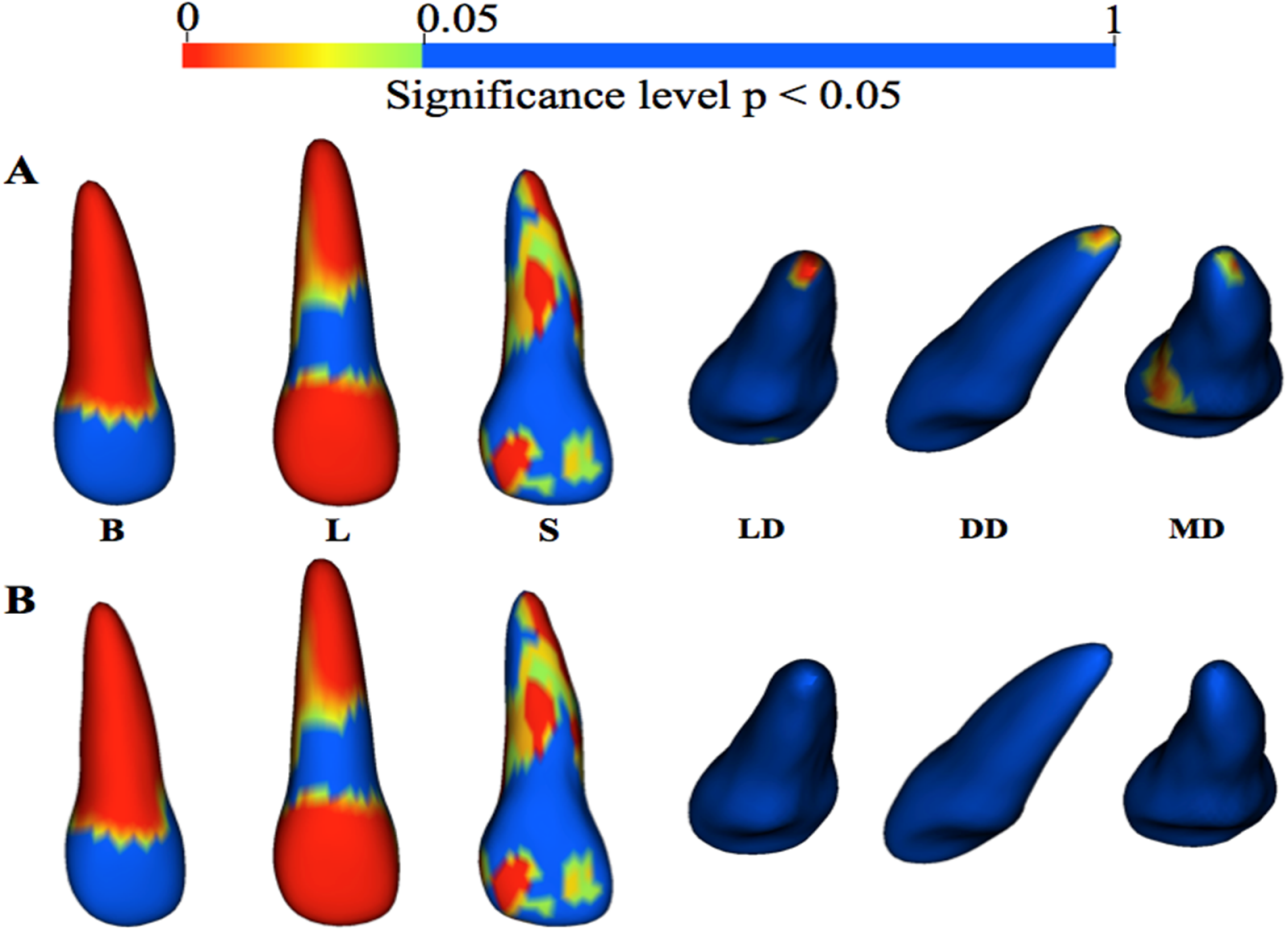
(A) Raw p-value (p < 0.05) color maps showing regions of statistically significant surface-to-surface differences for lateral incisor blunt (B), long (L), short (S), lingual dilaceration (LD), distal dilaceration (DD), and mesial dilaceration (MD) subgroup average models compared to the neutral average using Hotelling’s t-tests. (B) P-values corrected for false discovery rate at 0.05.

For the lateral incisor subgroup averages, raw p-value color maps localized the regions of statistically significant differences in root morphology for all groups compared to the neutral average model (p < 0.05). Raw p-value color maps for the lateral incisors are shown in Fig 9. The regions of significant morphological differences between the subgroup averages to the neutral average were as follows:

**Blunt**—wider at the cervical portion of the root and shorter at the apex
**Long**—longer and wider along entire root surface
**Short**—narrower at entire facial and mesial root and cervical 1/3 of distal of root
**Lingual dilaceration**—lingual deviation at root apex
**Distal dilaceration—**distal deviation at root apex
**Mesial dilaceration**—mesial deviation at root apex

With an FDR set at 0.05, significant differences for the lingual, mesial, and distal dilaceration average models compared to the neutral average (Fig 9) were not observed. Distribution of statistically significant regions for the blunt, conical, long, and short, as compared to the neutral average, were similar to what was shown by the raw p-value color maps.

## Discussion

Over the years, numerous studies have reported radiographic examinations of incisor root resorption and root shape using various imaging modalities, yet these investigations lacked well-defined diagnostic criteria for root morphology[4,20–24]. Because of recent advancements in radiology, CBCT multiplanar (axial, coronal, and sagittal) images have been shown to be superior to periapical radiography for the investigation of root resorption changes. A recent study by Ponder et al.[36] demonstrated that CBCT scans can be used for quantifications of lateral resorption defects and can measure external apical root resorption accurately. The present study quantifies precisely 3D incisor morphology using shape correspondence methodology.

The current study validated the construction of surface models of maxillary central and lateral incisors from CBCT images, with excellent intraclass correlation between repeated surface models for total number of points, triangles, surface area and volume. For 20 repeated models with thousands of points each, only one model showed a difference of 0.86 mm, which was located at a single surface point distance in the crown. This validation study indicates that the methodology presented is highly reproducible.

With visualization of the surface-to-surface distance color-coded maps, the smaller than 0.5 mm differences observed in constructed models would not affect the categorization of a particular tooth into a specific morphology subgroup. The excellent reproducibility using CBCT scans observed in this study corroborates the findings of Lund et al. and Sherrard et al.[25,26], respectively, comparing CBCT to dry skull measurements and periapical radiographs. Recent development with non-radiation imaging modalities such as dental magnetic resonance still require further protocol developments to improve the three-dimensional rendering of root morphology[37,38].

In the present study, statistically significant incisor root morphological differences were established when blunt, long, conical, and short roots were compared to the neutral sample. Only small dilacerations (distal, mesial, and lingual) were observed in this study sample; statistically significant differences in the morphology of the dilacerated roots compared to the neutral average could not be confirmed after correction for false discovery rate.

These data can be used as a possible predictor for root resorption during orthodontic treatment because root resorption susceptibility following the application of an orthodontic force can be associated with root resorption before treatment (shorter roots) [8,9], and root morphology, particularly conical roots[7–9]. Dilacerated roots as observed in the incisor root finite element models proposed by Kamble et al. and Oyama et al.[18,19] potentially led to an altered distribution of the stress when forces were applied to teeth during orthodontic tooth movement.

In the current study, we did not observe teeth with *bottle shaped* or *eroded* morphologies described previously in the 2D study by Mirabella and Årtun[21]. Their evaluation was based on adult patients, whereas adolescents were considered in the current study. Normal and abnormal root loading during the aging process may contribute to erosions and bottle or pipette shaped root apices. Such factors influencing non-orthodontically related resorptive changes have been reported to include missing teeth, periodontal disease, bruxism, and occlusal trauma[1,2,39,40]. Additional studies would be required to determine if this difference in observed morphology is due to 2D versus 3D visualization methods or if it is the result of changes in morphology with aging. Probably we did not observe teeth with bottle shaped or eroded morphologies also due to the sample size in the current study.

The description of variability in root morphology in the current study was derived from the calculated signed distance measurements comparing unaltered size of individual tooth models to the average. It is possible that long roots could respond to stress and loading similarly as neutral roots and the observed long root morphology in this study simply includes teeth of larger dimensions.

Shape correspondence also offers the opportunity of mapping correlations of biological markers with morphological variability, which was not undertaken in the present study. This technology will allow future studies to map the stages of disease progression in root resorption longitudinally and identify morphological variants or subtypes, which may explain the heterogeneity of clinical presentation.

Automatic continuous assessment of root morphology makes a quantification of root resorption progress possible during treatment. Such assessment can be expected to decrease inter-investigator variability as well as reduce artifacts introduced by measurements in cross-sectional slices. Finesse in the quantification of root morphology is integral to the phenotyping process. These improvements in shape analysis technique associated to adequate genotyping[3,6] have the potential for acquiring new knowledge on the pathophysiology of root resorption and laying the groundwork for novel therapeutic intervention.

The entire tooth structure (crown and root) was used in this study to allow for the best possible superimposition of the surface models and an accurate correspondence across the sample. Differences in crown morphology for males versus females and in certain ethnicities have been documented in the literature, as for example an average central incisor crown width of 8.9 ± 0.6 mm in males versus 8.7 ± 0.6 mm in females[41].

Overall, the results of this investigation indicate that 3D shape analysis can be applied to the study of maxillary incisor root morphology, which offers the ability to evaluate changes in root size and shape both across samples pre-treatment as well as pre- to post-treatment changes. Application of this morphological characterization in additional studies may allow improved understanding of factors affecting development of root shape or the influence of root morphology on root resorption.

Pre- and post-treatment surface models presumably would have identical crown morphologies for registration of the models (unless of course restorative work or enameloplasty was performed during treatment), allowing accurate surface-to-surface registration of the models. This results in reliable comparisons of length, volume, and overall root morphology. Furthermore, resorption levels for each morphology type could be compared to determine if a statistically significant difference in the level of post-treatment resorption can be observed across subgroups.

## Conclusions

1. Three-dimensional shape analysis using cone-beam tomography images is a reproducible method for the construction of maxillary central and lateral incisor surface models.
2. Three-dimensional shape analysis using CBCT images allows a novel and detailed phenotypic characterization of maxillary incisor root morphology.
3. Statistically significant differences in root morphology exist between subgroup averages of blunt, long, conical, and short central incisors compared to the neutral average.
